# The risk of second primary cancer after nasopharyngeal cancer: a systematic review

**DOI:** 10.1007/s00405-023-08144-0

**Published:** 2023-07-26

**Authors:** Fanni Svärd, Rasheed Omobolaji Alabi, Ilmo Leivo, Antti A. Mäkitie, Alhadi Almangush

**Affiliations:** 1 Department of Otorhinolaryngology, Kuopio University Hospital, Kuopio, Finland; 2 Research Program in Systems Oncology, Faculty of Medicine, University of Helsinki, Helsinki, Finland; 3 Department of Otorhinolaryngology, Head and Neck Surgery, Helsinki University Hospital and University of Helsinki, Helsinki, Finland; 4 Division of Ear, Nose and Throat Diseases, Department of Clinical Sciences, Intervention and Technology, Karolinska Institutet and Karolinska University Hospital, Stockholm, Sweden; 5 Department of Pathology, University of Helsinki, P.O. Box 21, 00014 Helsinki, Finland; 6 Institute of Biomedicine, Pathology, University of Turku, Turku, Finland

**Keywords:** Nasopharyngeal carcinoma, Second primary cancer, Head and neck cancer, Systematic review

## Abstract

**Purpose:**

Second primary cancers (SPCs) after nasopharyngeal cancer (NPC) are rare, but have an impact on the follow-up of this patient population. The aim of this study is to systematically review the literature to determine the prevalence and most typical sites of SPCs after NPC.

**Methods:**

We searched the databases of PubMed, Web of Science, and Scopus for articles on SPCs after NPC. The Preferred Reporting Items for Systematic Review and Meta-Analyses guidelines were followed.

**Results:**

This review includes data on 89 168 patients with NPC from 21 articles. The mean occurrence for SPCs was 6.6% and varied from 4.9% in endemic areas to 8.7% in non-endemic areas. The most frequent locations of SPCs were oral cavity, pharynx, nose and paranasal sinuses, esophagus and lung.

**Conclusion:**

There is an increased risk for a SPC after NPC management, especially in non-endemic areas. However, their mean rate is lower than after other head and neck carcinomas.

## Introduction

Nasopharyngeal carcinoma (NPC) is a relatively rare malignancy in most parts of the world, but the incidence is as much as 50–100 fold higher in ethnic Cantonese Chinese populations in Southern China and Southeast Asia, where it is endemic. Intermediately increased rates of NPC are found among native populations of other endemic areas, including Inuits and Aleuts in the Arctic, and populations in Northern Africa and parts of the Middle East. Populations in other areas of the world have lower rates of NPC, and such areas are thus considered non-endemic [1].

The geographical variability in incidence rates suggests different origins and risk factors for NPC. In endemic areas, Epstein–Barr virus and genetic factors are among established risk factors for NPC [1, 2]. Another well-known risk factor is the consumption of traditionally preserved food (particularly Chinese-style salted fish), whereas the role of occupational exposure to formaldehyde and wood dust in NPC development has recently been questioned [1]. The impact of tobacco smoking on the development of NPC has long been debated, but smoking is currently considered a proven risk factor for NPC in both high- and low-incidence areas [1, 2]. In addition, most NPCs are of the undifferentiated type that has a distinct biological behavior compared to squamous cell carcinoma, which is the most frequent type of carcinoma in other head and neck areas [1].

Patients with head and neck cancer have been proven to have an increased risk of second primary cancers (SPCs), especially in the upper aerodigestive tract [3, 4]. For example, the SPC rate after oral [5] or laryngeal [6] cancer has been estimated to be as high as 25% and 30%, respectively. However, studies on SPCs in patients with NPC are scarce. These studies have mainly been conducted in high-incidence countries. To date, SPCs after NPC have only been addressed as a minor part of two reviews [4, 7], and no review focused solely on NPC SPCs has been published.

The objective of this systematic review was to determine the average rate and typical location of SPCs in NPC survivors, and to compare their frequency to that of SPCs after other head and neck carcinomas.

## Materials and methods

**Fig. 1:**
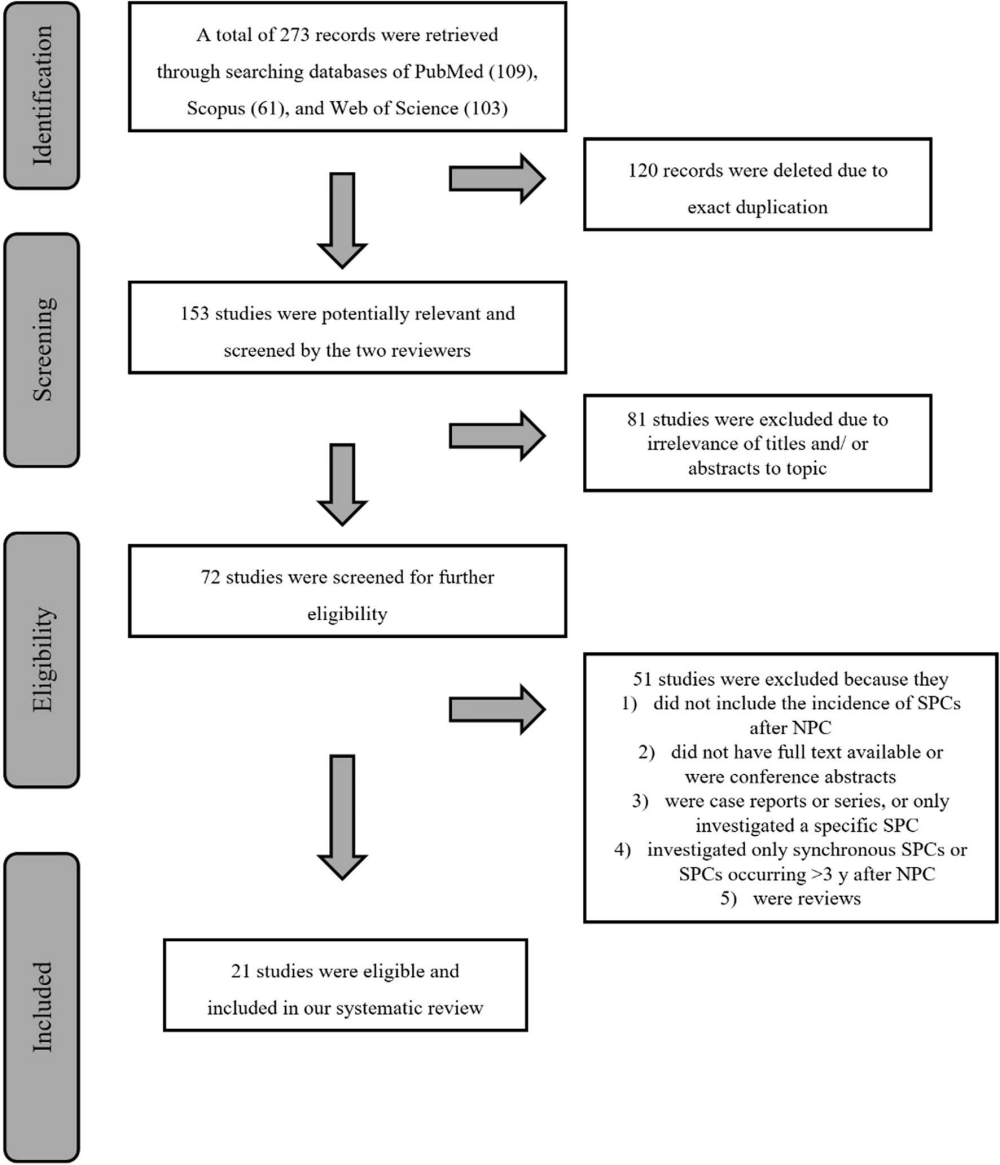
PRISMA flowchart: studies included and excluded along the different steps

The Preferred Reporting Items for Systematic Review and Meta-Analyses (PRISMA) method was used to conduct a systematic review of the current literature. The search was conducted from inception to November 2022 in the following databases: PubMed, Web of Science and Scopus (Fig. 1). Research Ethics Committee approval was not needed for this systematic literature search.

Our database search was developed by combining the following key words**:** "Nasopharyngeal Carcinoma" and "Second Primary". The search was limited to studies on humans, and English language literature. Two authors (F.S., R.A.) independently reviewed the extracted articles to exclude duplicates and irrelevant articles. In case of disagreement, a discussion was conducted to reach a consensus.

Studies were selected if they met the following criteria: (a) patients treated for NPC, and (b) information on the percentage of SPCs in the series. Our search identified 273 articles. After deletion of duplicates, a total of 153 articles was retrieved. Eighty-one of them were determined to be irrelevant based on their abstracts, leaving 72 for closer examination. As the objective of this review was to study the frequency of all possible SPCs, studies that had investigated only a specific type of SPC were excluded. The same applied to a study that had investigated only SPCs occuring more than three years after the treatment for NPC, a study that had only examined synchronous SPCs, and two reviews (Fig. 1). Finally, 21 articles were found to be eligible for the review, and these are summarized in Table 1.

**Table 1:** Characteristics of the included studies

Reference	Year	Country	Number of NPC patients	Frequency of SPC (%)	Most typical locations of SPC	Latency (years)
Ayan et al.	1996	Turkey	50	6.3	Mandible, Hodgkin’s lymphoma, gastric	Median 6.4
Wang et al.	2000	Taiwan	1549	2.5	Head and neck, gastric, leukemia	–
Cağlar et al.	2006	Turkey	41	2.4	Kidney	–
Kong et al.	2006	China	326	5.2	Oral cavity, lung	Mean 3.6
Scélo et al.	2007	Singapore, Canada, Europe^a^, Australia	8947	3.3 (Singapore 1.5, others 5.7)	Tongue, nose and nasal cavities, upper aerodigestive tract	–
Tsou et al.	2007	Taiwan	584	2.9	Upper aerodigestive tract, lung	–
Chen et al.	2008	Taiwan	23 629	3.0	Oral cavity, pharynx, major salivary gland	Mean 5.3
Sultan et al.	2010	USA	5043	7.4	Not specified	Median 5.3
Goggins et al.	2010	Hong Kong	1500	5.6	Tongue, nasal cavity, brain	Median 4.3
Cheuk et al.	2011	USA	59	8.5	Maxilla, esophagus, minor salivary gland	Mean 19.8
Liao et al.	2013	Taiwan	27,834	5.2	Not specified	–
Lin et al.	2014	Taiwan	10,299	5.6	Nasal, lung, oral cavity, hematological^b^	Mean 2.8
Chan et al.	2015	USA	3162	10.5	Oral cavity, pharynx, esophagus, nose, middle ear, lung	Latency groups: 0.5–1, 1–5, 5–12, > 12^c^
Ooft et al.	2016	Netherlands	1175	20.2	Pharynx, larynx	–
Zhao et al.	2016	China	527	2.3	Lung, tongue	Median 2.7
Chow et al.	2018	Hong Kong	759	6.7	Tongue, oropharynx	Median 5.8
Ben-Ami et al.	2020	Israel	42	9.5	Skull base, neck, CML, testis	Median 8.6
Chow et al.	2020	Hong Kong	3166	9.2	Oral cavity, oropharynx, paranasal sinuses, salivary gland, thyroid, skin, lung	Latency groups: < 5 (*n* = 86), 5–10 (*n* = 148), > 10 (*n* = 56)
Bay et al.	2021	Turkey	108	8.3	Oral cavity, pharynx	Median 15
Kebudi et al.	2021	Turkey	92	8.7	Tongue, pharynx	Median 13
Niu et al.	2022	China	276	6.5	Lung, head and neck	–

^a^Denmark, Finland, Iceland, Norway, Slovenia, Sweden, Spain

^b^NPC patients had many cases of gastrointestinal and genitourinary tract carcinomas but the risk was equal to healthy controls

^c^Increased risk for SPC at all sites in all latency groups, but the study did not report number of cases

The information was retrieved from each paper including the name of the first author, year of publication, country of the study, number of NPC patients, frequency of SPC, most typical sites for SPC, and latency for SPC (Table 1). In addition, smoking, alcohol comsumpion and EBV status were retrieved if reported (data not shown).

## Results

Our review includes a total of 89 168 patients from 21 articles during the years 1961–2017 [8–28]. It is noteworthy that there is partial overlap between the populations of some studies [3–9]. Five of the studies investigated only children and adolescents with NPC [8, 10, 17, 26, 27]. All but one study were retrospective by design [21]. Most of the studies were conducted in high-incidence areas (China, Hong Kong, Taiwan, Singapore). The average median follow-up time was 7.7 years.

The mean frequency of SPCs was 6.6% (range 1.5–20.2). In high-incidence NPC areas the mean rate of SPCs was 4.9%, whereas in low-incidence areas (Canada, Europe, United States of America) it was 8.7%. Seven studies had calculated a standardized incidence ratio (SIR) for SPC [9, 12, 14, 16, 20, 21, 23]. For all countries and cancer sites combined, the mean SIR of SPC was 2.0.

The main locations for the SPCs were oral cavity, pharynx, nose and paranasal sinuses, esophagus and lung. In studies that defined the locations more precisely, the most frequent location for the SPC in oral cavity was tongue [12, 16, 22, 23, 27, 29]. The most typical locations of SPCs were for the most part the same in all countries.

The median latency period of SPC varied from 2.7 to 15 years giving an average median of 7.7 years. However, instead of median value, four studies reported the mean latency ranging from 2.8 to 19.8 years. Six studies did not announce latency for SPC after NPC [9, 10, 12, 18, 21, 28] and two studies had divided latency into categories [20, 25].

There was some discrepancy in risk factors for SPC development. Some studies suggested the risk of SPC was increased if NPC was diagnosed and treated at a younger age (generally classified as under 40 or 50 years of age) [12, 14, 16, 19], but an equal number of studies found that the risk was increased in patients past this age [11, 21, 23, 25]. In addition, four studies suggested female sex as a risk factor for SPCs [14, 16, 19, 20]. Most studies provided no information on smoking, but all but one of these studies stated that a history of smoking was an independent predictive factor for SPC [13, 22, 23, 25]. Only Tsou et al. [13] included excessive alcohol consumption in their analysis finding no association with the SPC risk. EBV infection is a well-known risk factor for NPC, but none of the four studies that had included EBV status in their analyses found significant association between EBV and SPCs [9, 14, 19, 21].

## Discussion

Head and neck cancer survivors are known to be at a high risk of SPC [3, 4]. However, little is known about the risk of SPC after NPC treatment. In this systematic review, the mean SPC rate after NPC treatment was 6.6%. This is remarkably less than the rate of SPCs after head and neck cancers in general: a recent systematic review by Coca-Pelaz et al. [3]. including 456 130 patients from 61 articles reported a mean rate of 13.2%. In our study, the mean rate of SPCs was 4.9% in endemic areas of NPC and 8.7% inlow-incidence areas.

The lower rates of SPC after NPC than after other head and neck carcinomas may be due to their different mechanisms of carcinogenesis. In other head and neck carcinomas, the theory of field cancerization proposed by Slaughter et al. [30] is widely accepted for the development of SPCs. According to this theory, the upper aerodigestive tract mucosa accumulates genetic alterations after repeated carcinogenic exposure, such as smoking and alcohol consumption, and this results in the development of several independent malignant lesions. It seems that this theory can be partly applied to SPCs after NPC, as tobacco smoking has been proven to be a risk factor for also NPC, including both squamous cell carcinoma (SCC) -type virus-negative NPC and EBV-positive NPC via promoting EBV activation and, hence, viral carcinogenesis [31]. In our review, all but one of the studies that included information on smoking stated that a history of smoking was an independent risk factor for developing SPC [22, 23, 25]. Only Tsou et al. [13] did not find an increased risk for SPC in smokers versus nonsmokers. However, many endemic area studies stated that smoking is very common in these countries, and yet the mean rate of SPCs was generally lower in endemic areas than in Western countries. It is possible that the epidemiology of both NPC and SPCs differs in low- and high-incidence areas. This is supported by data indicating that the proportion of keratinizing SCC among NPCs is higher in low-incidence areas than in high-incidence areas [1, 12, 20]. In Western countries, smoking may play a greater role in the development of NPC and hence cause SPCs [20, 21]. In addition, higher prevalence of human papillomavirus (HPV) DNA can be seen in SCC-type NPC compared to the non-keratinizing types. However, the majority of SCC-type NPCs are both HPV and EBV negative [32].

In this systematic review, the main site for SPCs in virtually all countries was the upper aerodigestive tract, similar to other head and neck cancers [3, 4]. The most common locations, the oral cavity, pharynx, nose and paranasal sinuses, esophagus, and lung, are all susceptible to environmental factors, such as smoking. Moreover, many of them are located in the irradiation field.

Radiotherapy is the main line of treatment for NPC [2]. However, it causes major adverse effects that affect quality of life and is a risk factor for the development of SPCs [33]. Radiation-induced cancers are thought to develop many years after treatment [33]. In our review, the average median latency of SPC was 7.9 years, the median latency varying from 2.7 to 15 years. However, it is noteworthy that in many studies, the follow-up time was relatively short, given that secondary cancers may appear decades after treatment of the first cancer.

Some studies suggested that women have an increased risk for SPCs compared to men [14, 16, 19, 20], while other studies found no significant gender differences. Most studies did not investigate the risk of SPC in different subsites between sexes; however, Chan et al. [20] found the most significant difference in the oral cavity and pharynx, in which women were significantly more at risk for SPC than men with the most significant risk after a latency period of more than 5 years. This suggests that women may be more likely to develop SPCs after equal dosage of radiation therapy than men, which is in line with a review by Tubiana et al. [34]. However, the reason for this difference remains unclear.

Most studies included in this systematic review were conducted when the main line of treatment was conventional radiotherapy with or without chemotherapy, but some had used intensity modulated radiation therapy (IMRT) [22, 23, 25, 28]. IMRT has improved dose-delivery to NPC, but as a downside an increased area of normal tissue is exposed to low-dose radiation [35]. Some studies have indicated that IMRT increases the risk of radiation-induced cancers remarkably [36]. Of the studies in this review, Chow et al. [25] and Zhao et al. [22] compared the treatment modalities used and found that at least the 5-year incidence of SPCs among NPC patients treated with IMRT was concordant with that of the previous conventional 2-dimensional radiotherapy studies [22, 25]. Based on the locations of SPCs in the studies of this review it would seem that the main sites are in the near proximity of nasopharynx, but, e.g., the brain and temporal area were spared using IMRT instead of conventional radiotherapy.

Of the studies included in this review, only Kong et al. [11] and Zhao et al. [22] reported the histological subtypes of all SPCs. None of the SPCs were undifferentiated, which would support the theory of field cancerization by e.g. tobacco smoking or radiotherapy. However, it has been proposed that if field cancerization plays a major role, the relationship between the two cancers in question should be seen in both directions in the same extent [37]. There is an excess risk of NPC as SPC after other head and neck carcinomas, but it appears to be lesser than that of other head and neck carcinomas after NPC [4–7].

There was a discrepancy in whether older or younger patients had a higher risk of SPCs. Some studies found the risk of SPC to be increased if NPC was diagnosed or treated at a younger age (classified as < 40 or 50 years of age) [12, 14, 16, 19], whereas other studies suggested that the risk was increased in patients past this age [11, 13, 21, 23, 25]. It would make sense that the younger the person, the more time they would have to get another cancer. It is also possible that the lower baseline cancer risk in younger patients contributes to a higher excess risk, whereas the baseline risk for cancers in total increases with age. Moreover, some studies proposed that common genetic factors may play a role in SPC risk in younger patients. Interestingly, regarding laryngeal carcinoma, a study by Silén et al. [38] reported that SPCs in younger patients occurred at approximately the same age as in laryngeal carcinoma patients in general. However, the reason behind this remained unknown. The studies included in this systematic review did not compare the latency between younger and older patients, and this remains an interesting research area.

To our knowledge, this is the first systematic review on SPCs after NPC. However, this review has its limitations. It is noteworthy that the follow-up times of some studies were relatively short given that the latency of SPC, especially of SPCs related to radiation, may be as long as a decade or more. Most studies had used the criteria of SPC by Warren and Gates [39], but some had used other criteria, which may affect the results. For example, Singapore has a strict definition for SPC [12], which may explain why overall second cancer risk was decreased after NPC. However, the risk was still increased for cancers in the head and neck area. Many studies lacked information on the types of both NPCs and SPCs. Tsou et al. [13] found no significant differences in SPC development between NPC types. On the other hand, Ooft etl al. [21] showed that the risk of SPC after NPC is increased in the SCC type of NPC (the ones usually not related to EBV) in particular. These differing results may reflect demographical differences in NPC etiology between Taiwan and The Netherlands. Regarding all SPCs, it was mostly not stated whether SPC was SCC or, e.g., a sarcoma which has especially been linked to radiation [14, 40]. However, most SPCs have been stated to be SCC [41].

## Conclusion

There is an increased SPC risk after NPC, especially in non-endemic areas, but the risk is lower than after other head and neck carcinomas. At least some of this excess risk is likely due to treatment effects, but shared genetic and environmental risk factors may be involved. As the survival time of NPC patients has the potential to increase due to earlier diagnosis and improvements in treatment, the frequency of SPCs may increase and this needs to be considered in the follow-up of this patient population.

## Data Availability

Not applicable for this study.
